# Identification and characterization of immune-related lncRNAs and lncRNA-miRNA-mRNA networks of *Paralichthys olivaceus* involved in *Vibrio anguillarum* infection

**DOI:** 10.1186/s12864-021-07780-2

**Published:** 2021-06-15

**Authors:** Xianhui Ning, Li Sun

**Affiliations:** 1 CAS Key Laboratory of Experimental Marine Biology, Center for Ocean Mega-Science, Institute of Oceanology, Chinese Academy of Sciences, 7 Nanhai Road, 266071 Qingdao, China; 2 College of Marine Science and Engineering, Nanjing Normal University, 210023 Nanjing, Jiangsu, China; 3 Laboratory for Marine Biology and Biotechnology, Qingdao National Laboratory for Marine Science and Technology, Qingdao, China; 4 Co-Innovation Center for Marine Bio-Industry Technology of Jiangsu Province, 222005 Lianyungang, Jiangsu, China

**Keywords:** LncRNA, *Paralichthys olivaceus*, *Vibrio anguillarum*, Immune pathway, ceRNA network

## Abstract

**Background:**

Long non-coding RNAs (lncRNAs) structurally resemble mRNAs and exert crucial effects on host immune defense against pathogen infection. Japanese flounder (*Paralichthys olivaceus*) is an economically important marine fish susceptible to *Vibrio anguillarum* infection. To date, study on lncRNAs in flounder is scarce.

**Results:**

Here, we reported the first systematic identification and characterization of flounder lncRNAs induced by *V*. *anguillarum* infection at different time points. A total of 2,368 lncRNAs were identified, 414 of which were differentially expressed lncRNAs (DElncRNAs) that responded significantly to *V*. *anguillarum* infection. For these DElncRNAs, 3,990 target genes (named DETGs) and 42 target miRNAs (named DETmiRs) were identified based on integrated analyses of lncRNA-mRNA and lncRNA-miRNA expressions, respectively. The DETGs were enriched in a cohort of functional pathways associated with immunity. In addition to modulating mRNAs, 36 DElncRNAs were also found to act as competitive endogenous RNAs (ceRNAs) that regulate 37 DETGs through 16 DETmiRs. The DETmiRs, DElncRNAs, and DETGs formed ceRNA regulatory networks consisting of 114 interacting DElncRNAs-DETmiRs-DETGs trinities spanning 10 immune pathways.

**Conclusions:**

This study provides a comprehensive picture of lncRNAs involved in *V*. *anguillarum* infection. The identified lncRNAs and ceRNA networks add new insights into the anti-bacterial immunity of flounder.

**Supplementary Information:**

The online version contains supplementary material available at 10.1186/s12864-021-07780-2.

## Background

Long non-coding RNAs (lncRNAs) of more than 200 nucleotides (nt) structurally resemble mRNAs, but exhibit poor sequence conservation and cannot be translated into functional proteins [1, 2]. LncRNAs exert vital effects on multiple biological processes, including development, reproduction, metabolism, and immunity [3–6]. Unlike microRNAs (miRNAs), whose post-transcriptional regulation mechanisms have been well characterized [7], the functional mechanisms of lncRNAs remain to be fully elucidated. Evidences show that lncRNAs modulate the expression of genes in close genomic proximity and distant transcriptional regulators via *cis*- and *trans*-acting, respectively [8]. Moreover, recent studies have revealed that lncRNAs can act as miRNA sponges to modulate the expressions of target mRNAs through common miRNA response elements (MREs) [9–11]. This regulatory mechanism is known as competitive endogenous RNA (ceRNA) activity, which generates a regulatory network across the transcriptome as a whole [12]. LncRNA-mediated ceRNA activity has been shown to be strongly relevant to cancer pathogenesis and provide important diagnostic biomarkers and therapeutic targets [13–15].

In teleost fish, infection-associated lncRNAs have been reported in a number of species. For example, it has been shown that tilapia lncRNAs were induced by *Streptococcus agalactiae* [16], rainbow trout lncRNAs were induced by *Flavobacterium psychrophilum* [17], orange-spotted grouper lncRNAs were induced by *Pseudomonas plecoglossicida* [18], and Atlantic salmon lncRNAs were induced by virus (infectious salmon anemia virus) [19], bacteria (*Piscirickettsia salmonis*) [19], and parasite (*Caligus rogercresseyi*) [19, 20]. The ceRNA activity controlled by circular RNA (circRNA)-miRNA-mRNA has also been reported to be implicated in fish immune regulation, for example, circRNA-mediated ceRNA was involved in anti-grass carp reovirus response and anti-*Edwardsiella tarda* response in grass carp and Japanese flounder, respectively [21–23]. To date, studies on lncRNA-mediated ceRNA in fish with pathogen infection are scare, except a recent report showing that a lncRNA regulated antiviral responses in miiuy croaker via ceRNA mechanism [24].

Japanese flounder (*Paralichthys olivaceus*) is an economically important marine fish in north Asia [25]. Flounder culture has been severely threatened by vibriosis, one of the most frequent aquaculture diseases caused by *Vibrio* spp, in particular *Vibrio anguillarum* [26]. Studies showed that some protein-coding genes of *V. anguillarum*, such as *VAA* [27], *OmpK* [28] and *OmpR* [29], are able to induce the immune responses of T and B lymphocytes in founder. Recently, transcriptome and micro-transcriptome analyses revealed that *V*. *anguillarum* induced the expression of a large amount immune related genes and miRNAs in Japanese flounder [30, 31]. However, no study on flounder lncRNA has been documented.

In this study, we systematically investigated the lncRNA expression profiles of flounder during *V*. *anguillarum* infection at 3 different time points. We identified the differentially expressed lncRNAs (DElncRNAs) induced by *V*. *anguillarum*, examined the integrative expressions of lncRNA-mRNA and lncRNA-miRNA, analyzed the target genes (termed DETGs) and target miRNAs (termed DETmiRs) of DElncRNAs, and characterized the immune-related ceRNA networks of DElncRNA-DETmiR-DETG. Our study provides a global profile of lncRNAs in flounder associated with bacterial infection, which adds new insights into the immune response of teleost during bacterial infection. The immune-related lncRNA-miRNA-mRNA networks identified in this study also can serve as potential targets for future investigations on the molecular mechanism of fish immune defense against bacterial pathogens.

## Results

### Identification and sequence characterization of lncRNAs

**Fig. 1:**
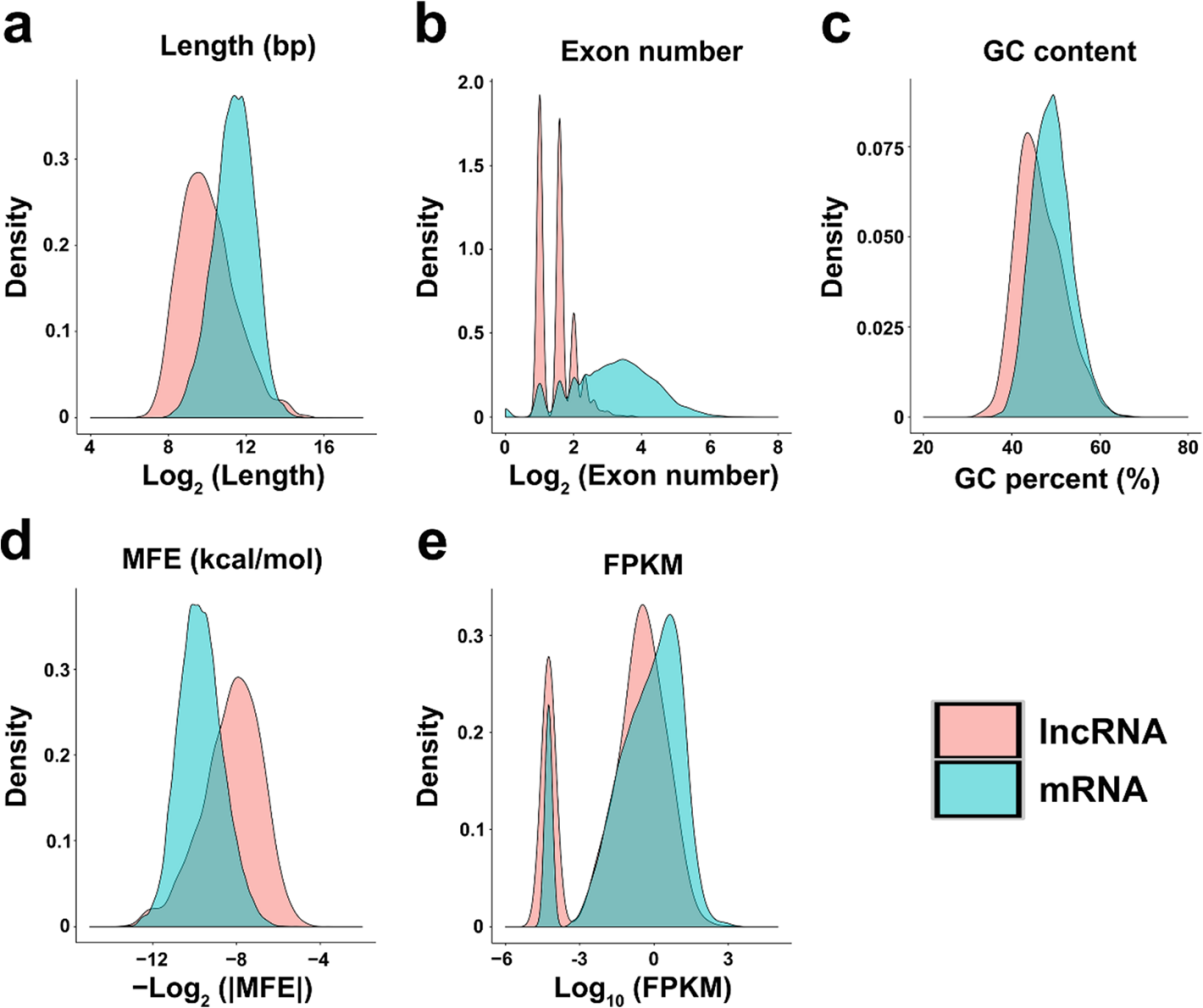
The features of lncRNAs versus mRNAs in Japanese flounder. **a** Sequence length. **b** The exon number contained in lncRNA or mRNA. **c** The guanine-cytosine (GC) content. **d** Minimum free energy (MFE). **e** Expression. FPKM, Fragments per kilobase of transcript per million mapped reads

In a previous study, transcriptome was conducted to examine the mRNA profiles of Japanese flounder infected with *V. anguillarum* for 6, 12, and 24 h [30]. In the present study, the dataset was analyzed for lncRNA expression, and 2,368 lncRNAs were identified. Based on their physical locations in the genome, the lncRNAs were classified into intergenic and genic lncRNAs. Specifically, 1823 (76.98 %) lncRNAs are intergenic lncRNAs that overlap no protein-coding loci in the genome of flounder, and 545 (23.02 %) lncRNAs are from genic regions that overlap protein-coding genes in the sense or antisense orientation. Sequence conservation analysis showed that only 40 (1.69 %) lncRNAs had hits with known lncRNAs in other species, including 15 hits in humans (*Homo sapiens*), 9 hits in mouse (*Mus musculus*), 9 hits in zebrafish (*Danio rerio*), 4 hits in rat (*Rattus norvegicus*), 2 hits in cattle (*Bos taurus*), and one hit in opossum (*Monodelphis domestica*) (Additional file 1). Compared with the mRNAs detected in the same samples in a previous study [30], the lncRNAs were shorter in length (1861 bp on average) than the mRNAs (3367 bp on average) (Fig. 1a). The exon number contained in lncRNAs ranged from 2 to 13, with an average of 1.59, which was less than that contained in the mRNAs (average of 3.60) (Fig. 1b). The guanine-cytosine (GC) content of the lncRNAs ranged from 32.22 to 66.80, with an average of 46.44, which was lower than that of the mRNAs (average of 49.11) (Fig. 1c). LncRNAs exhibited a lower absolute value of the minimum free energy (MFE), an index evaluating the stability of the secondary structure of RNAs, indicating that the secondary structures of the lncRNAs were less stable than that of the mRNAs (Fig. 1d). The average expression level of the lncRNAs was 3.09, which was lower than that of the mRNAs (average FPKM of 9.31) (Fig. 1e).

### Identification of *V. anguillarum*-induced lncRNAs

**Fig. 2:**
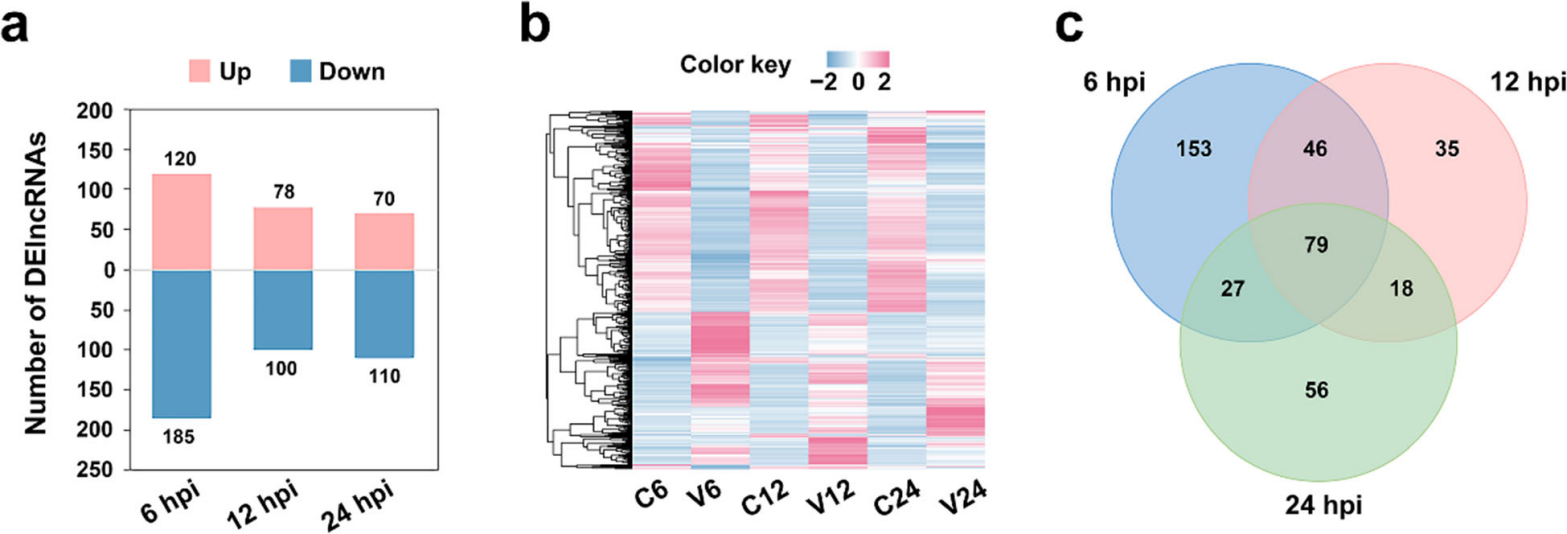
Differentially expressed lncRNAs (DElncRNAs) detected at different time points. **a** Number of DElncRNAs at 6, 12 and 24 h post-infection (hpi). “Up” and “Down” indicate up- and down-regulated expression. **b** The expression profiles of DElncRNAs in different groups at different time points. For convenience, the control groups and *V. anguillarum*-infected groups were labeled with the capital letters “C” and “V”, respectively. The number after “C”/ “V” indicates hpi. **c** Venn diagram showing overlapping DElncRNAs at different time points

After *V. anguillarum* challenge, 414 lncRNAs showed differential expressions at the 3 time points, and these lncRNAs were named DElncRNAs (Additional file 2). Specifically, at 6 h post-infection (hpi), 120 and 185 DElncRNAs were significantly up- and down-regulated, respectively; at 12 hpi, 78 and 100 DElncRNAs were significantly up- and down-regulated, respectively; at 24 hpi, 70 and 110 DElncRNAs were significantly up- and down-regulated, respectively (Fig. 2a, b). Seventy-nine (19.1 %) DElncRNAs were differentially expressed at all 3 time points (Fig. 2c). To validate the identified DElncRNAs, qRT-PCR was performed to determine the expressions of 8 DElncRNAs. The results showed high correlation coefficients (ranging from 0.86 to 0.99) with that of RNA-seq (Fig. S1), confirming the differential expression patterns of these DElncRNAs.

### Identification of the target genes (mRNAs) of DElncRNAs and functional enrichment based on DElncRNA-target interactions

In order to identify interactive lncRNA-mRNA pairs, lncRNA and mRNA co-expression and co-localization analyses were performed to predict *trans*- and *cis*-interactions, respectively. A total of 7,140 mRNAs were found to exhibit significantly strong correlations (|*r*| > 0.9, and *p* < 0.05) with DElncRNAs in expression. Of these putative interacting mRNAs, 3,975 were differentially expressed after *V. anguillarum* infection, 3,922 of which were physically far away from DElncRNAs. These 3,922 genes were considered as *trans*-differentially expressed target genes of DElncRNAs (*trans*-DETGs). Genomic location analysis showed that 59 DElncRNAs were located near 278 mRNAs, 68 of these mRNAs were differentially expressed after *V. anguillarum* infection and were considered as *cis*-DETGs of DElncRNAs. Additionally, 53 of the 68 *cis*-DETGs were strongly correlated (|*r*| > 0.9, and *p* < 0.05) in expression with their respective DElncRNAs. In total, 3,990 DETGs (Additional file 2) were identified for the 414 DElncRNAs.

**Fig. 3:**
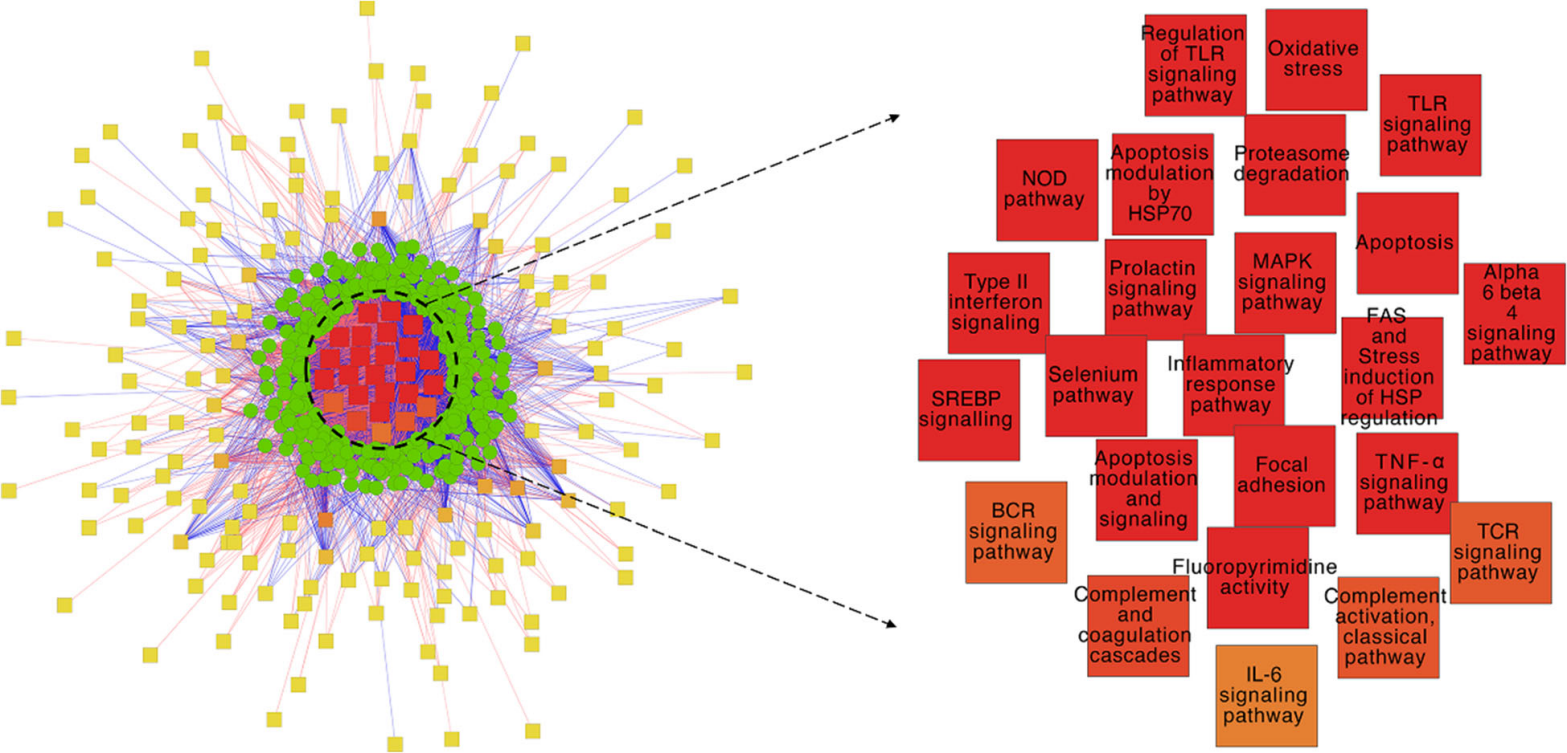
Functional enrichment network and the highly enriched pathways of DElncRNAs. DElncRNAs are indicated by green nodes. The pathways are indicated by squares colored from yellow to red according to their cumulative enrichment values (from low to high)

To gain insight into the biological processes in which the DElncRNAs were involved, functional enrichment network was constructed based on DElncRNA-DETG interactions and functional enrichment of the DETGs (Fig. 3). In the network, 25 pathways were highly enriched, at least 20 of which were associated with immunity, including TLR signaling pathway, TNF-α signaling pathway, NOD pathway, inflammatory response pathway, IL-6 signaling pathway, type II interferon signaling, complement activation pathway, complement and coagulation cascades, apoptosis, FasL and stress induction of HSP regulation, and BCR and TCR signaling pathways (Fig. 3).

### Identification of the target miRNAs of DElncRNAs

In a previous study of micro-transcriptome analysis, 1,218 miRNAs were identified in flounder [31]. In the present study, all the 1,218 miRNAs were predicted to be the targets of DElncRNAs. These miRNAs were subjected to correlation analysis with the expressions of DElncRNAs. The result showed that 74 miRNAs were significantly and negatively correlated with 164 DElncRNAs in expression. Further analysis was conducted with the 74 miRNAs based on their responses to *V. anguillarum* infection, and only the miRNAs with differential expression after *V. anguillarum* infection were selected. Finally, 42 miRNAs were identified as differentially expressed target miRNAs of DElncRNAs and were named DETmiRs (Additional file 2), whose expressions were both significantly regulated by *V. anguillarum* and significantly correlated (*p* < 0.05) with DElncRNAs expressions in a negative manner. The expression patterns of six pairs of DElncRNA-DETmiR, i.e., pol-lnc735-miR-21-y, pol-lnc735-pol-miR-21-3p, pol-lnc491-pol-miR-21-3p, pol-lnc131-pol-miR-n199-3p, pol-lnc163-pol-miR-n199-3p, and pol-lnc491-miR-221-x, were validated by qRT-PCR (Fig. S2). The results showed that in each pair, the expressions of DElncRNA and DETmiR were significantly (*p* < 0.05) and negatively correlated, with correlation coefficient *r* ranging from − 0.83 to − 0.96 (Fig. S2).

### Construction of immune-related ceRNA networks of interactive DElncRNA-DETmiR-DETG

**Fig. 4:**
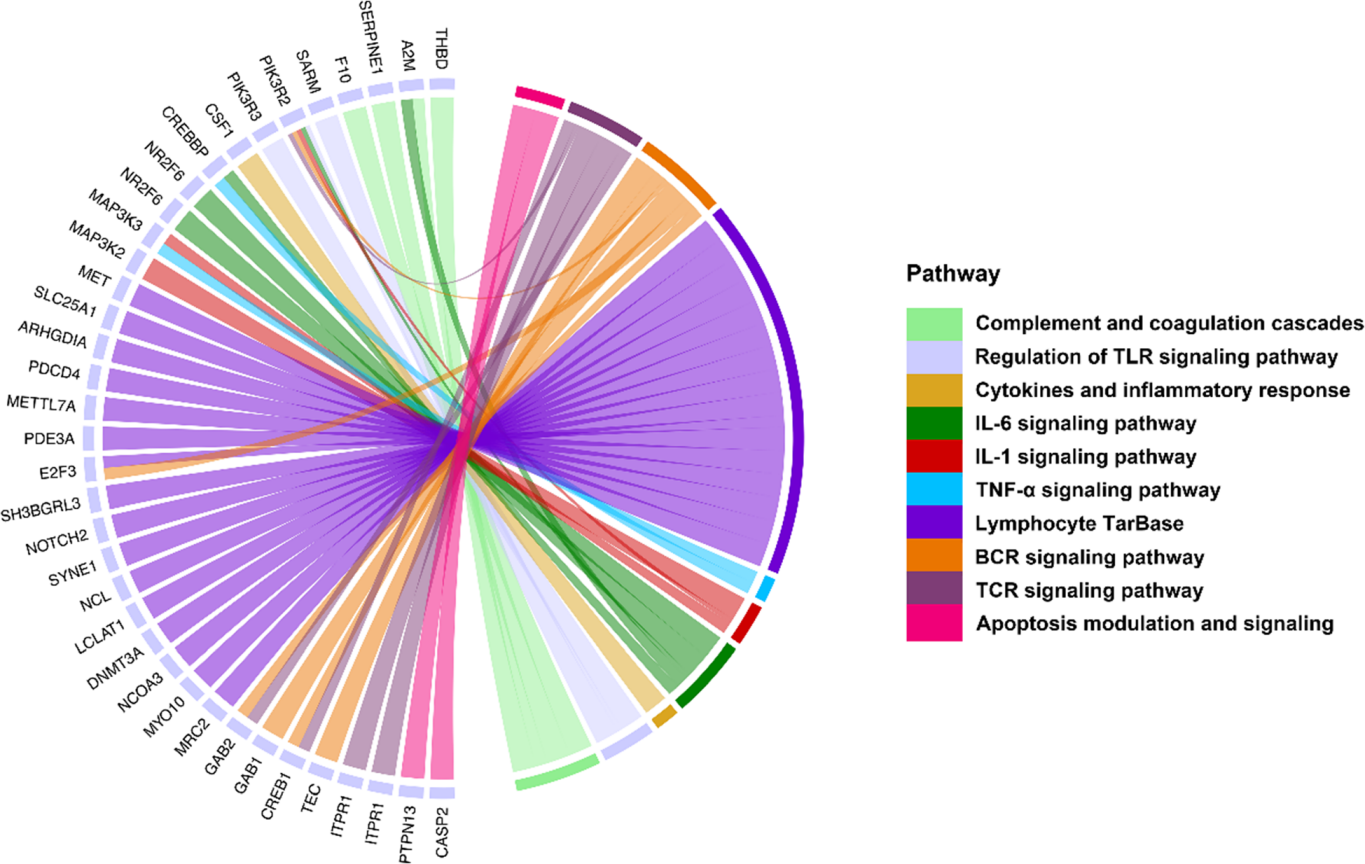
Immune-related pathways enriched in the competitive endogenous DETGs of DElncRNAs

**Fig. 5:**
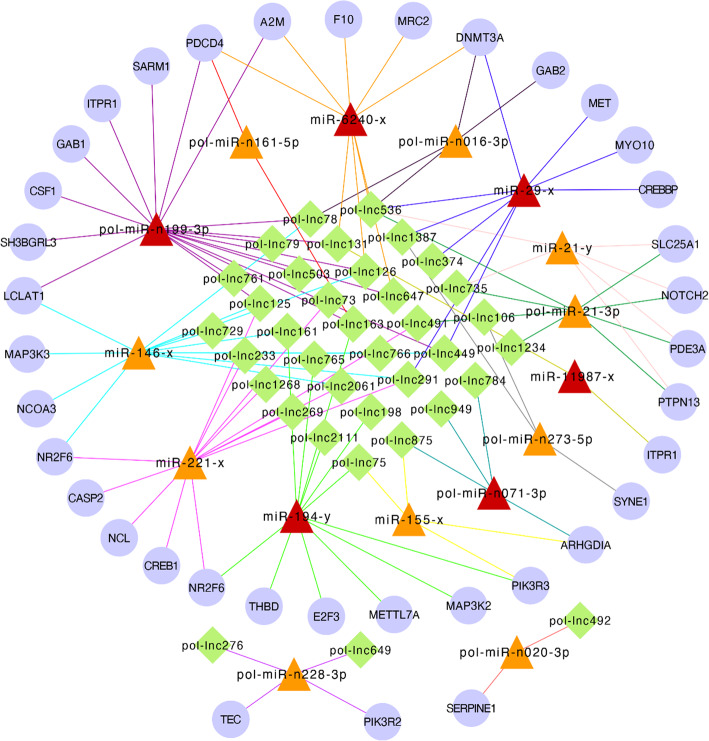
Immune-related DElncRNA-DETmiR-DETG ceRNA networks. DElncRNA, differentially expressed lncRNA. DETmiR, differentially expressed target miRNA of DElncRNA. DETG, differentially expressed target gene of DElncRNA. CeRNA, competitive endogenous RNA. The round nodes indicate DETGs. The triangle nodes indicate DETmiRs, of which the key miRNAs identified in a miRNA transcriptome analysis of flounder infected with *Vibrio anguillarum* are labeled in red. The diamond nodes indicate DElncRNAs. “pol-lnc” indicates Japanese flounder lncRNAs

Integrated analyses of the interactions of DElncRNAs-DETmiRs and DEmiRs-DETGs, as well as the competitions of DElncRNAs-DETGs through MREs, were performed. As a result, 87 DElncRNAs, 28 DETmiRs, and 609 DETGs with interactive relationships were identified. Functional enrichment analysis revealed that these 609 competitive endogenous DETGs (ceDETGs) were involved in 10 immune-related pathways, including the signaling pathways of TLR, IL-6, IL-1, TNF-α, BCR, and TCR, as well as complement and coagulation cascades, apoptosis modulation and signaling, cytokines and inflammatory response, and lymphocyte TarBase (miR-targeted genes in leukocyte) (Fig. 4). The expression patterns of three DETGs (*THBD*, *SERPINE1*, and *F10*) involved in the pathway of complement and coagulation cascades were validated by qRT-PCR, which showed high correlations with that of RNA-seq (*r* ranging from 0.89 to 0.93) (Fig. S3). To gain insights into the role of the DElncRNA-DETmiR-DETG trinities in the immune response to *V. anguillarum* infection, an immune-related ceRNA network was constructed based on functional enrichment of the ceDETGs. The network consisted of 36 DElncRNAs, 16 DEmiRs, and 37 DETGs, which formed 114 interacting trinities that spanned 10 pathways associated with immunity (Fig. 5). The DETGs in the ceRNA trinities included *SARM* (sterile alpha and armadillo motif-containing protein), *A2M* (alpha-2-macroglobulin), *F10* (coagulation factor Χ), *CSF1* (colony stimulating factor 1), *CREB1* (cAMP-responsive element-binding protein 1), *CREBBP* (CREB-binding protein), *MAP3K2* (mitogen-activated protein kinase kinase kinase 2), *MAP3K3* (mitogen-activated protein kinase kinase kinase 3), *CASP2* (caspase-2), *PTPN13* (protein tyrosine phosphatase-N13), *GAB1* (GRB2-associated-binding protein 1), *GAB2* (GRB2-associated-binding protein 2), *PDCD4* (programmed cell death protein 4), and *SLC25A1* (solute carrier family 25 member 1). In the ceRNA networks, six of the DEmiRs, i.e., pol-miR-n199-3p, pol-miR-n071-3p, miR-6240-x, miR-29-x, miR-11,987-x, and miR-194-y, were key immune-related DEmiRs identified in a previous study [31].

## Discussion

In this study, we examined the lncRNAs of Japanese flounder induced by *V. anguillarum*. We found that more than 98 % of the flounder lncRNAs had no orthologs in other species, which concurred with the notion that most lncRNAs lack primary sequence conservation [32]. Compared with mRNAs, lncRNAs exhibited lower MFE values, suggesting a flexibility in their secondary structures, which may facilitate their binding to miRNAs and mRNAs [33]. Other characteristics of flounder lncRNAs, including exon number, GC content, and expression level, were similar to that observed in the lncRNAs of yellow croaker, rainbow trout, Atlantic salmon, tilapia, and tongue sole [16, 17, 19, 34, 35]. However, the average length of founder lncRNAs (1861 bp) was longer than that of the lncRNAs of zebrafish (1113 bp), tilapia (764 bp), rainbow trout (400 bp), and Atlantic salmon (400 bp) [19, 34, 36, 37]. Since we used only three individual samples in each group at each time point, there is a possibility that some of the characteristics observed in our study may be different if the sample size is increased.

In this study, a total of 414 DElncRNAs were identified to be affected by *V. anguillarum* infection in a time-dependent fashion, with more DElncRNAs detected in the early infection stage. LncRNAs play an important role in host immune defense against pathogen infection [38–40]. Congruously, we found that flounder DElncRNAs, by targeting DETGs, were highly involved in the pathways associated with immunity. The enrichment of the pathways of TLR signaling and complement activation indicated that *V. anguillarum* stimulated the pathogen recognition process and initiated host immune response. The pathogen recognition process was also found to play an important role in our previous study on *V. anguillarum*-induced core immune genes of founder [30]. Pathways associated with inflammation and apoptosis were also strongly enriched, suggesting an involvement of these responses in the clearance of the invading pathogen as reported previously [41, 42]. In rainbow trout and tilapia, pathogen-induced host lncRNAs were shown to be engaged in adaptive immunity, such as TCR signaling and MHC protein complex [16, 17]. In our study, we found that the DETGs of DElncRNAs were enriched in BCR and TCR signaling pathways. These results suggest that fish lncRNAs are able to stimulate both innate and adaptive immune responses, which likely provide more efficient protections against pathogen infection. In mammals, lncRNAs are known to regulate host immunity by serving as ceRNAs to modulate mRNA expression [43–45]. In our study, immune-related ceRNA networks were found to be formed by 36 DElncRNAs and their corresponding DETmiRs and DETGs involved in 10 immune pathways, including pathogen recognition, inflammation, apoptosis, and adaptive immunity response, which are discussed below.

### Pathogen recognition and killing

In this study, pol-lnc78 was localized in the immune-related ceRNA network and targeted pol-miR-n199-3p, which regulated SARM, a newly identified TLR adaptor. In mammals, SARM is known to be a negative regulator of immune response through the TLR signaling pathway, and suppress LPS- and poly (I:C)-mediated AP-1 activation during pathogen infection [46–48]. In fish, *SARM* expression was down-regulated by LPS, and over-expression of *SARM* inhibited GCRV (grass carp reovirus) triggered IFN-I response [49]. In accordance with the previous reports, we found that *SARM* was enriched in the TLR signaling pathway and significantly reduced during *V. anguillarum* infection at all 3 time points, suggesting a persisted activation of TLR signaling and the pathogen recognition process. In addition to *SARM*, *A2M* and *F10* were also identified in our study as the targets of pol-lnc126 (via pol-miR-n199-3p/miR-6240-x) and pol-lnc131 (via miR-6240-x), respectively. *A2M* has a common evolutionary origin with complement components C3 and C4, and was shown to contribute to bacteriostatic activity in amphioxus [50–52]. F10 is involved in antibacterial infection through initiating the coagulation cascade [53], and induced by both bacterial (*Aeromonas hydrophila*) and fungal (*Aphanomyces invadans*) infections in fish [54]. Taken together, these observations indicate that in flounder, *V. anguillarum* infection induces ceRNA trinities that likely promote pathogen recognition and bacterial killing.

### NF-κB-regulated inflammation response

In this study, we found that pol-lnc79, pol-lnc163, and pol-lnc449 competitively targeted pol-miR-n199-3p against *CSF1*, and pol-lnc291, pol-lnc449, and pol-lnc1387 acted as ceRNAs that regulated *CREBBP* by sponging miR-29-x. Pol-miR-n199-3p and miR-29-x were identified as the key miRNAs induced by *V. anguillarum* infection in our previous micro-transcriptome analysis of the same samples [31]. CSF1 is a cytokine highly implicated in inflammation [55, 56]; CREBBP is a crucial cofactor that binds numerous transcription factors, e.g., human CREBBP cooperates with NF-κB to regulate IL-6 promoter [57, 58]. MAP3K1-3 also exerts a crucial effect on NF-κB activation [59, 60]. In mice, MAP3K3 affects both TNF- and IL-1-induced NF-κB activation [61]. Moreover, MAP3K3 was reported to activate NF-κB through the lncRNA-MALAT1-miR-424-*MAP3K3* axis [62]. In our study, pol-lnc198, pol-lnc765, and pol-lnc2111 sponged miR-194-y to regulate *MAP3K2* associated IL-1 signaling pathway, and pol-lnc126 sponged miR-146-x to regulate *MAP3K3* associated IL-1 and TNF-α signaling pathways. Together, these results suggest that lncRNA-miRNA-mRNA trinities are involved in the regulation of NF-κB-mediated inflammatory response.

### Apoptosis

Four lncRNAs, i.e., pol-lnc73, pol-lnc233, pol-lnc491, and pol-lnc2061, were found to regulate *CASP2* by competitively binding miR-221-x, and four other lncRNAs, i.e., pol-lnc491, pol-lnc536, pol-lnc735, and pol-lnc1234, regulated *PTPN13* by competitively binding pol-miR-21-3p or miR-21-y. CASP2 is an initiator for apoptosis execution and involved in bacteria-induced immune defense in striped murrel [63]. PTPN13 is known to inhibit Fas-induced apoptosis, and its down-regulation significantly increased the survival of human papillomavirus infected patients with squamous cell carcinoma [64, 65]. In this study, we found that *CASP2* and *PTPN13* were the targets of lncRNAs and down-regulated during infection. The suppression of these target genes suggests that they may be part of the host defensive response against pathogen, or the consequence of bacterial manipulation of the host immune system.

### Adaptive immunity

BCR signaling is pivotal for the activation of B cells, and TCR recognition of pathogen-derived peptides is a hallmark of the adaptive immunity [66, 67]. In this study, we found that pol-lnc78, pol-lnc125, and pol-lnc291/pol-lnc491 regulated *GAB2*, *GAB1*, and *CREB1*, respectively, by sponging the target miRNAs, and *GAB1/2* and *CREB1* were involved in the signaling pathways of BCR and TCR. GAB1/2 are key adaptor/scaffolding factors mediating signal transductions down-stream of BCR and TCR [68], and CREB1 is a CREB/ATF family protein controlling the transcription of various viruses (human T-lymphotropic virus, herpes simplex virus, Epstein-Barr virus, and cytomegalovirus) in humans [69]. These observations indicate that in flounder, *V. anguillarum* probably induces T and B cell-mediated adaptive immunity via lncRNA-regulated ceRNA axes.

## Conclusions

This study identified systematically the lncRNAs of Japanese flounder induced by *V*. *anguillarum*. A total of 414 DElncRNAs were detected, which target 3,990 DETGs and 42 DETmiRs. Thirty-six DElncRNAs compete with DETmiRs to regulate DETGs and form immune related ceRNA networks. These observations indicate that in teleost, bacteria-induced immune response is controlled by complicated regulatory networks involving non-coding RNAs and ceRNAs. The results of our study add new insights into the anti-microbial defense of flounder and provide a valuable dataset for future studies on the immune mechanism and disease control of flounder based on non-coding RNAs and ceRNAs.

## Methods

### LncRNA identification

LncRNA analysis was performed with the previously reported raw RNA sequencing data of 18 spleen libraries of flounder infected with and without (control) *V*. *anguillarum* [30]. In this previous study [30], Japanese flounder (purchased from a local commercial fish farm in Shandong Province, China) were injected with *V. anguillarum* C312 (named group V) or PBS (control, named group C); at 6, 12, and 24 h post infection (hpi), three fish from each group were collected and euthanized with tricaine methanesulfonate (Sigma, St. Louis, USA); spleen was then collected from the fish and used for RNA sequencing [30]. In the present study, the software fastp (v0.12.4) was used to filter the raw reads to obtain high quality reads, which were then mapped to the reference genome of Japanese flounder (GenBank project accession PRJNA369269) using TopHat 2 (v2.0.3.12) [70] as previously reported [30]. Cufflinks (v2.2.1) [71] was used to reconstruct transcripts from the mapped reads as previously reported [30]. The transcripts with the length of more than 200 bp were analyzed using the software coding-non-coding-index (CNCI, v2), a powerful tool to effectively distinguish protein-coding and non-coding sequences independent of known annotations, to filter out the protein-coding mRNAs [72]. The software coding potential calculator (CPC) was used to evaluate the protein-coding potential of the remaining non-coding sequences [73]. Moreover, the non-coding sequences were also searched against the UniProtKB/SwissProt database, and the sequences with blast hits of *E*-values < 1e-6 were removed. Finally, the transcripts assessed to have no protein-coding potential and have no hits in the database were identified as lncRNAs of Japanese flounder and named pol-lnc1 to pol-lnc2368. The conservation of the lncRNAs was evaluated by aligning with the lncRNAs of other species in database NONCODE v5.0 [74].

### Differential expression analysis

The abundance of each lncRNA was counted using RSEM [75], and the expression level was normalized with FPKM (Fragments Per Kilobase of transcript per Million mapped reads) [76]. The R package edgeR (v3.12.1) (http://www.r-project.org/) was applied to conduct differential expression analysis. False discovery rate (FDR) < 0.05 and fold change (FC) in expression > 2 (log_2_|FC| > 1) were set as the threshold to identify the differentially expressed lncRNAs (DElncRNAs).

### Experimental validation

To verify the DElncRNAs obtained by RNA-seq, quantitative real-time reverse transcription-PCR (qRT-PCR) was employed to test the expression of eight randomly selected DElncRNAs at 6, 12, and 24 hpi (three fish/time point) with the collected spleen tissues that had been used for RNA-seq in the previous study [30]. The expression profiles of DETmiRs were evaluated by qRT-PCR with the collected spleen tissues that had been used for micro-transcriptome in the previous study [31]. Similarly, three DETGs (*THBD*, *SERPINE1*, and *F10*) of DElncRNAs were validated for expression by qRT-PCR. The PCR reactions were performed with SYBR Premix Ex TaqII (TaKaRa, Dalian, China) in QuantStudio 3 Real-Time PCR Systems (Thermo Fisher Scientific, CA, USA) according to the manufacturer’s protocol. At the end of each reaction, the melting curve analysis was conducted to verify the specific of PCR product. The expression levels of lncRNAs, mRNAs and miRNAs was evaluated with 2^−ΔΔCt^ comparative Ct method [77]. Correlations between qRT-PCR and RNA-seq results, as well as between DElncRNAs and their corresponding DETmiRs, were analyzed using cor.test in R (v3.5.2). The primer sequences were listed in Table 1.

**Table 1:** Summary of primers used for qRT-PCR in this study

RNA	Primer	Sequence (5’ to 3’)
Pol-lnc1387	1387-f	TGAAGTCCTGCTGTCATCGC
	1387-r	GTGGTAAAAACCCCGGTCCT
Pol-lnc2180	2180-f	CAGATGACCCGCAACTCCAT
	2180-r	TTCCTCGTCACATCGACACC
Pol-lnc1234	1234-f	CGTCTAATGAGCAGCCGAGT
	1234-r	AATGAGAGTGAGAGCGGTGC
Pol-lnc2360	2360-f	CGATTCGTCCTGAGCAGGTT
	2360-r	TCGTTGTCACACAGGTCGAG
Pol-lnc73	73-f	TCAATCTTATCCTTCCGTTCC
	73-r	TTCCTCAGCGTTTCCTTTG
Pol-lnc922	922-f	CCGTCTGTACGAGAATGTCC
	922-r	TCTGAACCACCTGAGCCAC
Pol-lnc449	449-f	TAACACGCCCCACACTAACC
	449-r	GTTCCCCTCCCCCTCTACAT
Pol-lnc503	503-f	CAGACGCTTGACGGTTGTTG
	503-r	TACGTACGCTGAATACCGCC
Pol-lnc735	735-f	GGTGGTAAATGGCGTCGTGT
	735-r	CCTGGTGGAGCAGAAGGAGTT
Pol-lnc491	491-f	GCGGATACTTGATTTCCCACC
	491-r	TCCAATCCCAGTGTCAGTTGTCT
Pol-lnc131	131-f	AATGTCCTCCCTCATCCTAAAGC
	131-r	TTCATCCGTAAACAACCCAAGTC
Pol-lnc163	163-f	TTTATCTGACAGCGTTACAGCACC
	163-r	CAACATGACATTTGGACAACCTTC
THBD	thbd-f	CACGACTCCTGTCAGCTTGT
	thbd-r	GGAGACTGTTCTCTGCGCTT
SERPINE1	sne-f	AGGAAGGGGTGGAGATAGCC
	sne-r	TTAGAGATGGCACCTGCTGTG
F10	f10-f	AAACAGTGAGTGAGTGGGCA
	f10-r	GGGATCAATGTGTGCTCGTCT
TUBA	tuba-f	TGACATCACAAACGCCTGCTTC
	tuba-r	GCACCACATCTCCACGGTACAG
miR-21-y	21y-fz	GTCGTATCCAGTGCAGGGTCCGAGGTATTCGCACTGGATACGACACAGCC
	21y-f	CGCGCGAGACAACAGTCTGTA
	21y-r	AGTGCAGGGTCCGAGGTATT
pol-miR-21-3p	21p-fz	GTCGTATCCAGTGCAGGGTCCGAGGTATTCGCACTGGATACGACGACAGC
	21p-f	CGCGCGACAACAGTCTGAAG
	21p-r	AGTGCAGGGTCCGAGGTATT
pol-miR-n199-3p	199-fz	GTCGTATCCAGTGCAGGGTCCGAGGTATTCGCACTGGATACGACGCCAGC
	199-f	CGCGCAACACTGGTTTGTAA
	199-r	AGTGCAGGGTCCGAGGTATT
miR-221-x	221-fz	GTCGTATCCAGTGCAGGGTCCGAGGTATTCGCACTGGATACGACACAGAA
	221-f	GCGACCTGGCATACAATGTAGAT
	221-r	AGTGCAGGGTCCGAGGTATT
5 s	5 s-fz	CGGTCTCCCATCCAAGTA
	5 s-f	CCATACCACCCTGAACAC
	5 s-r	CGGTCTCCCATCCAAGTA

### Target genes identification

Based on the *trans-* and *cis-*acting of lncRNAs, the target genes for DElncRNAs were predicted by co-expression and co-location analysis [78]. The co-expression analysis was performed using cor.test in R (v3.5.2) based on the lncRNA expression data in this study and the mRNA expression data in a previous study with the same samples [30]. All of the 2,368 lncRNAs identified in this study and the 43,494 mRNAs identified previously [30] were used in the analysis. The expression of mRNAs was counted using RSEM (v1.2.19) and normalized across libraries using FPKM (Fragments Per Kilobase of transcript per Million mapped reads) method as described previously [30]. The genes correlated (correlation coefficient *r* > 0.9 or *r* < − 0.9, and *p* < 0.05) with DElncRNAs in expression were identified as the candidate *trans*-regulated target genes [79], which were then subjected to analysis of differential expression induced by *V. anguillarum* using R (v3.5.2). As reported previously [30], the exact negative binomial test in the R package edgeR (v3.12.1) (http://www.r-project.org/) was used to conduct pairwise comparison between group C and group V at each time point, and the threshold for significant difference was set as false discovery rate (FDR) < 0.05 and log2|FC| >1. The genes that both correlated with DElncRNAs expressions and differentially induced by *V. anguillarum* were subjected to distance evaluation. Finally, the genes that were differentially expressed, correlated with DElncRNAs, and located far from DElncRNAs (> 10 kb) were considered as *trans*-regulated target genes of DElncRNAs (named *trans*-DETGs). The co-location analysis was conducted according to the physical distance between the lncRNAs and potential target genes in the genome of flounder. The genes that physically overlapped or were near (< 10 kb) to DElncRNAs were identified as candidate *cis*-regulated target genes, which were then analyzed for differential expression induced by *V. anguillarum* using R (v3.5.2). The genes that were both co-located with DElncRNAs and differentially expressed after *V. anguillarum* infection were considered as *cis*-regulated target genes (named *cis*-DETGs). All the *trans*- and *cis*-regulated target genes were termed differentially expressed target genes of DElncRNAs (named DETGs).

### Functional enrichment network analysis

Enrichment analysis and visualization (ENViz), a Cytoscape plug-in, is generally used to predict biological functions engaged by ncRNAs [80]. In this study, ENViz of Cytoscape (v3.7.1) was applied to the analysis of DElncRNAs and their DETGs to construct the lncRNA-pathway enrichment networks. The pathways with a cumulative enrichment cutoff value > 3 were displayed and colored from yellow to red according to their cumulative enrichment values ranging from low to high, and the pathway with a cumulative enrichment cutoff value > 250 was considered a highly enriched pathway.

### Identification of the target miRNAs of DElncRNAs

Three softwares, i.e., RNAhybrid (v2.1.2) + svm_light (v6.01), Miranda (v3.3a), and TargetScan (v7.0), were employed to predict the interacting miRNAs of DElncRNAs using 1,218 miRNAs of flounder identified in a previous study with the same samples [31] and the DElncRNAs identified in this study. The common results predicted by the three algorithms were identified as candidate target miRNAs. For these candidate target miRNAs, R (v3.5.2) was employed to detect the differentially expressed miRNAs induced by *V. anguillarum*, and cor.test in R (v3.5.2) was used to detect the differentially expressed miRNAs that negatively correlated (*r* < − 0.7, *p* < 0.05) with their DElncRNAs based on the lncRNA data in this study and the miRNA data in a previous study with the same samples [31]. The candidate target miRNAs that were both differentially expressed during *V. anguillarum* infection and negatively correlated with DElncRNAs expressions were identified as the differentially expressed target miRNAs of DElncRNAs and were named DETmiRs.

### ceRNA network construction

Integrated analysis was conducted with several datasets, including the lncRNA expression data in this study and the mRNA expression data and miRNA expression data in previous studies with the same samples [30, 31]. Based on the ceRNA hypothesis, i.e., lncRNAs act as miRNA sponges to relieve the expression inhibition of miRNAs on the target mRNAs [12], the interacting DElncRNA-miRNA-mRNA networks were identified as follows: (1) detection of the negatively correlated DElncRNA-DETmiR pairs; (2) detection of the negatively correlated DETG-DEmiR pairs; (3) detection of the positively correlated DElncRNA-DETG pairs, in which, the DElncRNA and DETG of each pair share the same miRNA response elements (MREs). To be stringent, the hypergeometric cumulative distribution function test was applied to obtain the significantly enriched DElncRNA-DETG pairs that targeted the common miRNAs, with the threshold of *p* < 0.05. After above procedures, the remaining were the intertwined DElncRNA-DETmiR-DETG ceRNA trinities. The immune-related ceRNA network was constructed with Cytoscape (v3.7.1) [81] using the competitive endogenous DETGs enriched in the immune-related pathways and their corresponding DElncRNA-DETmiR in the intertwined DElncRNA-DETmiR-DETG trinities.

## Supplementary Information

**Additional file 1.** Summary of the conservation of Japanese flounder lncRNAs in other species.

**Additional file 2.** List of DElncRNAs, DETGs, and DETmiRs.

**Additional file 3: Figure S1.** Validation of DElncRNAs by qRT-PCR. The expression patterns of eight DElncRNAs were tested by qRT-PCR, and the results were compared with that obtained by RNA-sEq. The results are shown as means ± standard deviation (*N* = 3). Correlations between qRT-PCR and RNA-seq are indicated by correlation coefficient *r*.

**Additional file 4: Figure S2.** Validation of DElncRNA-DETmiR pairs by qRT-PCR. The expression patterns of six pairs of DElncRNA-DETmiR were tested by qRT-PCR. Correlations between DElncRNAs and corresponding DETmiRs are indicated by correlation coefficient *r* and *p* values.

**Additional file 5: Figure S3.** Validation of DETGs by qRT-PCR. The expression patterns of three DETGs involved in the pathway of complement and coagulation cascades were tested by qRT-PCR, and the results were compared with that obtained by RNA-sEq. The results are shown as means ± standard deviation (*N* = 3). Correlations between qRT-PCR and RNA-seq are indicated by correlation coefficient *r*.

## Data Availability

The raw data of RNA sequencing and small RNA sequencing are available at the Sequence Read Archive (SRA) in NCBI with the accession number of PRJNA554220 and SRP241633, respectively. The datasets generated during this study are included in the article and its additional files.

## Abbreviations

LncRNAs: Long non-coding RNAs
DElncRNAs: Differentially expressed lncRNAs
ceRNAs: Competitive endogenous RNAs
MREs: miRNA response elements
DETGs: Target genes of DElncRNAs
DETmiRs: Target miRNAs of DElncRNAs
SARM: Sterile alpha and armadillo motif-containing protein
A2M: Alpha-2-macroglobulin
F10: Coagulation factor Χ
CSF1: Colony stimulating factor 1
CREB1: cAMP-responsive element-binding protein 1
CREBBP: CREB-binding protein
MAP3K2: Mitogen-activated protein kinase kinase kinase 2
MAP3K3: Mitogen-activated protein kinase kinase kinase 3
CASP2: Caspase-2
PTPN13: Protein tyrosine phosphatase-N13
GAB1: GRB2-associated-binding protein 1
GAB2: GRB2-associated-binding protein 2
PDCD4: Programmed cell death protein 4
SLC25A1: Solute carrier family 25 member 1

## References

[1] Kapranov P, Cheng J, Dike S, Nix DA, Duttagupta R, Willingham AT, Stadler PF, Hertel J, Hackermuller J, Hofacker IL (2007). RNA maps reveal new RNA classes and a possible function for pervasive transcription. Science.

[2] Ulitsky I, Shkumatava A, Jan CH, Sive H, Bartel DP (2011). Conserved function of lincRNAs in vertebrate embryonic development despite rapid sequence evolution. Cell.

[3] Sarangdhar MA, Chaubey D, Srikakulam N, Pillai B (2018). Parentally inherited long non-coding RNA Cyrano is involved in zebrafish neurodevelopment. Nucleic Acids Res.

[4] Golicz AA, Bhalla PL, Singh MB (2018). lncRNAs in plant and animal sexual reproduction. Trends Plant Sci.

[5] Wang P, Xu JF, Wang YJ, Cao XT (2017). An interferon-independent lncRNA promotes viral replication by modulating cellular metabolism. Science.

[6] Du M, Yuan L, Tan X, Huang DD, Wang XJ, Zheng Z, Mao XX, Li XR, Yang L, Huang K (2017). The LPS-inducible lncRNA Mirt2 is a negative regulator of inflammation. Nat Commun.

[7] Bagga S, Bracht J, Hunter S, Massirer K, Holtz J, Eachus R, Pasquinelli AE (2005). Regulation by let-7 and lin-4 miRNAs results in target mRNA degradation. Cell.

[8] Ponting CP, Oliver PL, Reik W (2009). Evolution and functions of long noncoding RNAs. Cell.

[9] Wang Y, Xu ZY, Jiang JF, Xu C, Kang JH, Xiao L, Wu MJ, Xiong J, Guo XC, Liu HQ (2013). Endogenous miRNA sponge lincRNA-RoR regulates Oct4, Nanog, and Sox2 in human embryonic stem cell self-renewal. Dev Cell.

[10] Shan YJ, Ma J, Pan Y, Hu JL, Liu B, Jia L (2018). LncRNA SNHG7 sponges miR-216b to promote proliferation and liver metastasis of colorectal cancer through upregulating GALNT1. Cell Death Dis.

[11] Liang LL, Xu JC, Wang M, Xu GR, Zhang N, Wang GZ, Zhao YF (2018). LncRNA HCP5 promotes follicular thyroid carcinoma progression via miRNAs sponge. Cell Death Dis.

[12] Salmena L, Poliseno L, Tay Y, Kats L, Pandolfi PP (2011). A ceRNA hypothesis: the Rosetta Stone of a hidden RNA language?. Cell.

[13] Wang H, Huo XS, Yang XR, He J, Cheng LJ, Wang N, Deng X, Jin HJ, Wang N, Wang C (2017). STAT3-mediated upregulation of lncRNA HOXD-AS1 as a ceRNA facilitates liver cancer metastasis by regulating SOX4. Mol Cancer.

[14] Chen DL, Lu YX, Zhang JX, Wei XL, Wang F, Zeng ZL, Pan ZZ, Yuan YF, Wang FH, Pelicano H (2017). Long non-coding RNA UICLM promotes colorectal cancer liver metastasis by acting as a ceRNA for microRNA-215 to regulate ZEB2 expression. Theranostics.

[15] Abdollahzadeh R, Daraei A, Mansoori Y, Sepahvand M, Amoli MM, Tavakkoly-Bazzaz J (2019). Competing endogenous RNA (ceRNA) cross talk and language in ceRNA regulatory networks: A new look at hallmarks of breast cancer. J Cell Physiol.

[16] Luo HL, Yang HZ, Lin Y, Zhang YD, Pan CY, Feng PF, Yu YL, Chen XH (2017). LncRNA and mRNA profiling during activation of tilapia macrophages by HSP70 and *Streptococcus agalactiae* antigen. Oncotarget.

[17] Paneru B, Al-Tobasei R, Palti Y, Wiens GD, Salem M (2016). Differential expression of long non-coding RNAs in three genetic lines of rainbow trout in response to infection with *Flavobacterium psychrophilum*. Sci Rep.

[18] Zhang BB, Luo G, Zhao LM, Huang LX, Qin YX, Su YQ, Yan QP (2018). Integration of RNAi and RNA-seq uncovers the immune responses of *Epinephelus coioides* to L321_RS19110 gene of *Pseudomonas plecoglossicida*. Fish Shellfish Immunol.

[19] Tarifeño-Saldivia E, Valenzuela-Miranda D, Gallardo-Escárate C (2017). In the shadow: The emerging role of long non-coding RNAs in the immune response of Atlantic salmon. Dev Comp Immunol.

[20] Tarifeño-Saldivia E, Valenzuela-Miranda D, Gallardo-Escárate C (2018). Comparative analysis of long non-coding RNAs in Atlantic and Coho salmon reveals divergent transcriptome responses associated with immunity and tissue repair during sea lice infestation. Dev Comp Immunol.

[21] Wang M, Jiang S, Wu W, Yu F, Chang WG, Li PF, Wang K (2018). Non-coding RNAs function as immune regulators in teleost fish. Front Immunol.

[22] Xiu YJ, Jiang GP, Zhou S, Diao J, Liu HJ, Su BF, Li C (2019). Identification of potential immune-related circrna-mirna-mrna regulatory network in intestine of *Paralichthys olivaceus* during *Edwardsiella tarda* infection. Front Genet.

[23] Liu B, Yuan R, Liang Z, Zhang TT, Zhu M, Zhang X, Geng W, Fang P, Jiang MS, Wang ZY (2019). Comprehensive analysis of circRNA expression pattern and circRNA-mRNA-miRNA network in *Ctenopharyngodon idellus* kidney (CIK) cells after grass carp reovirus (GCRV) infection. Aquaculture.

[24] Chu Q, Xu TJ, Zheng WW, Chang RJ, Zhang L (2020). Long noncoding RNA MARL regulates antiviral responses through suppression miR-122-dependent MAVS downregulation in lower vertebrates. PLoS Pathog.

[25] Seikai T (2002). Flounder culture and its challenges in Asia. Rev Fish Sci.

[26] Egidius E (1987). Vibriosis - pathogenicity and pathology - a Review. Aquaculture.

[27] Xing J, Xu HS, Tang XQ, Sheng XZ, Zhan WB (2019). A DNA vaccine encoding the VAA gene of *Vibrio anguillarum* induces a protective immune response in flounder. Front Immunol.

[28] Xing J, Zhang ZQ, Luo KK, Tang XQ, Sheng XZ, Zhan WB (2020). T and B lymphocytes immune responses in flounder (*Paralichthys olivaceus*) induced by two forms of outer membrane protein K from *Vibrio anguillarum*: Subunit vaccine and DNA vaccine. Mol Immunol.

[29] Zhou XJ, Xing J, Tang XQ, Zhan WB (2018). Evaluation of bivalent vaccines candidates among VAA, OmpK and OmpR from *Vibrio anguillarum* in flounder (*Paralichthys olivaceus*). Dev Comp Immunol.

[30] Ning XH, Sun L (2020). Gene network analysis reveals a core set of genes involved in the immune response of Japanese flounder (*Paralichthys olivaceus*) against *Vibrio anguillarum* infection. Fish Shellfish Immunol.

[31] Ning XH, Sun L (2020). Micro-transcriptome analysis reveals immune-related microrna regulatory networks of *Paralichthys olivaceus* induced by *Vibrio anguillarum* infection. Int J Mol Sci.

[32] Khorkova O, Hsiao J, Wahlestedt C (2015). Basic biology and therapeutic implications of lncRNA. Adv Drug Deliv Rev.

[33] Dorn GW, Matkovich SJ (2014). Menage a Trois intimate relationship among a microRNA, long noncoding RNA, and mRNA. Circ Res.

[34] Li BJ, Jiang DL, Meng ZN, Zhang Y, Zhu ZX, Lin HR, Xia JH (2018). Genome-wide identification and differentially expression analysis of lncRNAs in tilapia. BMC Genom.

[35] Xu HG, Cao L, Sun B, Wei YL, Liang MQ (2019). Transcriptomic analysis of potential “lncRNA-mRNA” interactions in liver of the marine teleost *Cynoglossus semilaevis* fed diets with different DHA/EPA ratios. Front Physiol.

[36] Pauli A, Valen E, Lin MF, Garber M, Vastenhouw NL, Levin JZ, Fan L, Sandelin A, Rinn JL, Regev A (2012). Systematic identification of long noncoding RNAs expressed during zebrafish embryogenesis. Genome Res.

[37] Núñez-Acuña G, Détrée C, Gallardo-Escárate C, Gonçalves AT (2017). Functional diets modulate lncRNA-coding RNAs and gene interactions in the intestine of rainbow trout *Oncorhynchus mykiss*. Mar Biotechnol.

[38] Fitzgerald KA, Caffrey DR (2014). Long noncoding RNAs in innate and adaptive immunity. Curr Opin Immunol.

[39] Zhang Y, Cao XT (2016). Long noncoding RNAs in innate immunity. Cell Mol Immunol.

[40] Basavappa M, Cherry S, Henao-Mejia J (2019). Long noncoding RNAs and the regulation of innate immunity and host-virus interactions. J Leukoc Biol.

[41] Hacker G (2018). Apoptosis in infection. Microbes Infect.

[42] Wu RH, Sheng XZ, Tang XQ, Xing J, Zhan WB (2018). Transcriptome analysis of flounder (*Paralichthys olivaceus*) gill in response to lymphocystis disease virus (LCDV) infection: novel insights into fish defense mechanisms. Int J Mol Sci.

[43] Song YX, Sun JX, Zhao JH, Yang YC, Shi JX, Wu ZH, Chen XW, Gao P, Miao ZF, Wang ZN (2017). Non-coding RNAs participate in the regulatory network of CLDN4 via ceRNA mediated miRNA evasion. Nat Commun.

[44] Wu XS, Wang F, Li HF, Hu YP, Jiang L, Zhang F, Li ML, Wang XA, Jin YP, Zhang YJ (2017). LncRNA-PAGBC acts as a microRNA sponge and promotes gallbladder tumorigenesis. Embo Reports.

[45] Xu HN, Jiang Y, Xu XQ, Su XP, Liu Y, Ma YM, Zhao Y, Shen ZY, Huang B, Cao XT (2019). Inducible degradation of lncRNA Sros1 promotes IFN-gamma-mediated activation of innate immune responses by stabilizing Stat1 mRNA. Nat Immunol.

[46] Carty M, Goodbody R, Schroder M, Stack J, Moynagh PN, Bowie AG (2006). The human adaptor SARM negatively regulates adaptor protein TRIF-dependent Toll-like receptor signaling. Nat Immunol.

[47] Carty M, Bowie AG (2019). SARM: From immune regulator to cell executioner. Biochem Pharmacol.

[48] Peng J, Yuan QA, Lin B, Panneerselvam P, Wang XW, Luan XL, Lim SK, Leung BP, Ho B, Ding JL (2010). SARM inhibits both TRIF- and MyD88-mediated AP-1 activation. Eur J Immunol.

[49] Yan NN, Su JG, Yang CR, Rao YL, Feng XL, Wan QY, Lei CZ (2015). Grass carp SARM1 and its two splice variants negatively regulate IFN-I response and promote cell death upon GCRV infection at different subcellular locations. Dev Comp Immunol.

[50] Sottrup-Jensen L, Stepanik TM, Kristensen T, Lonblad PB, Jones CM, Wierzbicki DM, Magnusson S, Domdey H, Wetsel RA, Lundwall A (1985). Common evolutionary origin of alpha 2-macroglobulin and complement components C3 and C4. Proc Natl Acad Sci.

[51] Armstrong PB, Quigley JP (1999). Alpha2-macroglobulin: an evolutionarily conserved arm of the innate immune system. Dev Comp Immunol.

[52] Pathirana A, Diao M, Huang SB, Zuo LL, Liang YJ (2016). Alpha 2 macroglobulin is a maternally-derived immune factor in amphioxus embryos: New evidence for defense roles of maternal immune components in invertebrate chordate. Fish Shellfish Immunol.

[53] Chen JW, Li XJ, Li L, Zhang T, Zhang Q, Wu FM, Wang DY, Hu HZ, Tian CL, Liao DS (2019). Coagulation factors VII, IX and X are effective antibacterial proteins against drug-resistant Gram-negative bacteria. Cell Res.

[54] Arasu A, Kumaresan V, Sathyamoorthi A, Arasu MV, Al-Dhabi NA, Arockiaraj J (2016). Coagulation profile, gene expression and bioinformatics characterization of coagulation factor X of striped murrel *Channa striatus*. Fish Shellfish Immunol.

[55] Ho JL, Reed SG, Wick EA, Giordano M (1990). Granulocyte-macrophage and macrophage colony-stimulating factors activate intramacrophage killing of Leishmania mexicana amazonensis. J Infect Dis.

[56] Grayfer L, Hanington PC, Belosevic M (2009). Macrophage colony-stimulating factor (CSF-1) induces pro-inflammatory gene expression and enhances antimicrobial responses of goldfish (*Carassius auratus* L.) macrophages. Fish Shellfish Immunol.

[57] Spooren A, Kooijman R, Lintermans B, Van Craenenbroeck K, Vermeulen L, Haegeman G, Gerlo S (2010). Cooperation of NFkappaB and CREB to induce synergistic IL-6 expression in astrocytes. Cell Signal.

[58] Zhang QZ, Shi KC, Yoo D (2016). Suppression of type I interferon production by porcine epidemic diarrhea virus and degradation of CREB-binding protein by nsp1. Virology.

[59] Lee FS, Peters RT, Dang LC, Maniatis T (1998). MEKK1 activates both IκB kinase α and IκB kinase β. Proc Natl Acad Sci.

[60] Zhao Q, Lee FS (1999). Mitogen-activated protein kinase/ERK kinase kinases 2 and 3 activate nuclear factor-κB though IκB kinase-α and IκB kinase-β. J Biol Chem.

[61] Yang JH, Lin Y, Guo ZJ, Cheng J, Huang J, Deng L, Liao W, Chen Z, Liu Z, Su B (2001). The essential role of MEKK3 in TNF-induced NF-kappaB activation. Nat Immunol.

[62] Li MM, Dong CX, Sun B, Lei HZ, Wang YL, Gong YB, Sun LL, Sun ZW (2019). LncRNA-MALAT1 promotes tumorogenesis of infantile hemangioma by competitively binding miR-424 to stimulate MEKK3/NF-kappaB pathway. Life Sci.

[63] Kumaresan V, Ravichandran G, Nizam F, Dhayanithi NB, Arasu MV, Al-Dhabi NA, Harikrishnan R, Arockiaraj J (2016). Multifunctional murrel caspase 1, 2, 3, 8 and 9: Conservation, uniqueness and their pathogen-induced expression pattern. Fish Shellfish Immunol.

[64] Bamberg A, Redente EF, Groshong SD, Tuder RM, Cool CD, Keith RC, Edelman BL, Black BP, Cosgrove GP, Wynes MW (2018). Protein tyrosine phosphatase-n13 promotes myofibroblast resistance to apoptosis in idiopathic pulmonary fibrosis. Am J Respir Crit Care Med.

[65] Harris G, Bossler A (2010). PTPN13 expression correlates with survival in HPV + HNSCC. Otolaryngol Head Neck Surg.

[66] Pageon SV, Tabarin T, Yamamoto Y, Ma Y, Nicovich PR, Bridgeman JS, Cohnen A, Benzing C, Gao Y, Crowther MD (2016). Functional role of T-cell receptor nanoclusters in signal initiation and antigen discrimination. Proc Natl Acad Sci.

[67] Yang J, Reth M (2016). Receptor dissociation and B-cell activation. Curr Top Microbiol Immunol.

[68] Hibi M, Hirano T (2000). Gab-family adapter molecules in signal transduction of cytokine and growth factor receptors, and T and B cell antigen receptors. Leuk Lymphoma.

[69] Chia YL, Ng CH, Lashmit P, Chu KL, Lew QJ, Ho JP, Lim HL, Nissom PM, Stinski MF, Chao SH (2014). Inhibition of human cytomegalovirus replication by overexpression of CREB1. Antiviral Res.

[70] Kim D, Pertea G, Trapnell C, Pimentel H, Kelley R, Salzberg SL (2013). TopHat2: accurate alignment of transcriptomes in the presence of insertions, deletions and gene fusions. Genome Biol.

[71] Trapnell C, Roberts A, Goff L, Pertea G, Kim D, Kelley DR, Pimentel H, Salzberg SL, Rinn JL, Pachter L (2012). Differential gene and transcript expression analysis of RNA-seq experiments with TopHat and Cufflinks. Nat Protoc.

[72] Sun L, Luo HT, Bu DC, Zhao GG, Yu KT, Zhang CH, Liu YN, Chen RS, Zhao Y (2013). Utilizing sequence intrinsic composition to classify protein-coding and long non-coding transcripts. Nucleic Acids Res.

[73] Kong L, Zhang Y, Ye ZQ, Liu XQ, Zhao SQ, Wei L, Gao G (2007). CPC: assess the protein-coding potential of transcripts using sequence features and support vector machine. Nucleic Acids Res.

[74] Zhao Y, Li H, Fang SS, Kang Y, Wu W, Hao YJ, Li ZY, Bu DC, Sun NH, Zhang MQ (2016). NONCODE 2016: an informative and valuable data source of long non-coding RNAs. Nucleic Acids Res.

[75] Li B, Dewey CN (2011). RSEM: accurate transcript quantification from RNA-Seq data with or without a reference genome. BMC Bioinformatics.

[76] Trapnell C, Williams BA, Pertea G, Mortazavi A, Kwan G, van Baren MJ, Salzberg SL, Wold BJ, Pachter L (2010). Transcript assembly and quantification by RNA-Seq reveals unannotated transcripts and isoform switching during cell differentiation. Nat Biotechnol.

[77] Livak KJ, Schmittgen TD (2001). Analysis of relative gene expression data using real-time quantitative PCR. Methods.

[78] Liu LB, Xiao QH, Gilbert ER, Cui ZF, Zhao XL, Wang Y, Yin HD, Li DY, Zhang HH, Zhu Q (2018). Whole-transcriptome analysis of atrophic ovaries in broody chickens reveals regulatory pathways associated with proliferation and apoptosis. Sci Rep.

[79] Lyu KX, Li Y, Xu Y, Yue HJ, Wen YH, Liu TS, Chen SY, Liu QH, Yang WQ, Zhu XL (2020). Using RNA sequencing to identify a putative lncRNA-associated ceRNA network in laryngeal squamous cell carcinoma. RNA Biol.

[80] Steinfeld I, Navon R, Creech ML, Yakhini Z, Tsalenko A (2015). ENViz: a Cytoscape App for integrated statistical analysis and visualization of sample-matched data with multiple data types. Bioinformatics.

[81] Shannon P, Markiel A, Ozier O, Baliga NS, Wang JT, Ramage D, Amin N, Schwikowski B, Ideker T (2003). Cytoscape: a software environment for integrated models of biomolecular interaction networks. Genome Res.

