# Supplementary material for: Identification and characterization of immune-related lncRNAs and lncRNA-miRNA-mRNA networks of Paralichthys olivaceus involved in Vibrio anguillarum infection
Source: BMC Genomics. 2021 Jun 15;22:447. doi: 10.1186/s12864-021-07780-2 (PMC8204505; doi:10.1186/s12864-021-07780-2)

**Fig. S1.** Validation of DElncRNAs by qRT-PCR. The expression patterns of eight DElncRNAs were tested by qRT-PCR, and the results were compared with that obtained by RNA-seq. The results are shown as means ± standard deviation (N = 3). Correlations between qRT-PCR and RNA-seq are indicated by correlation coefficient *r*.


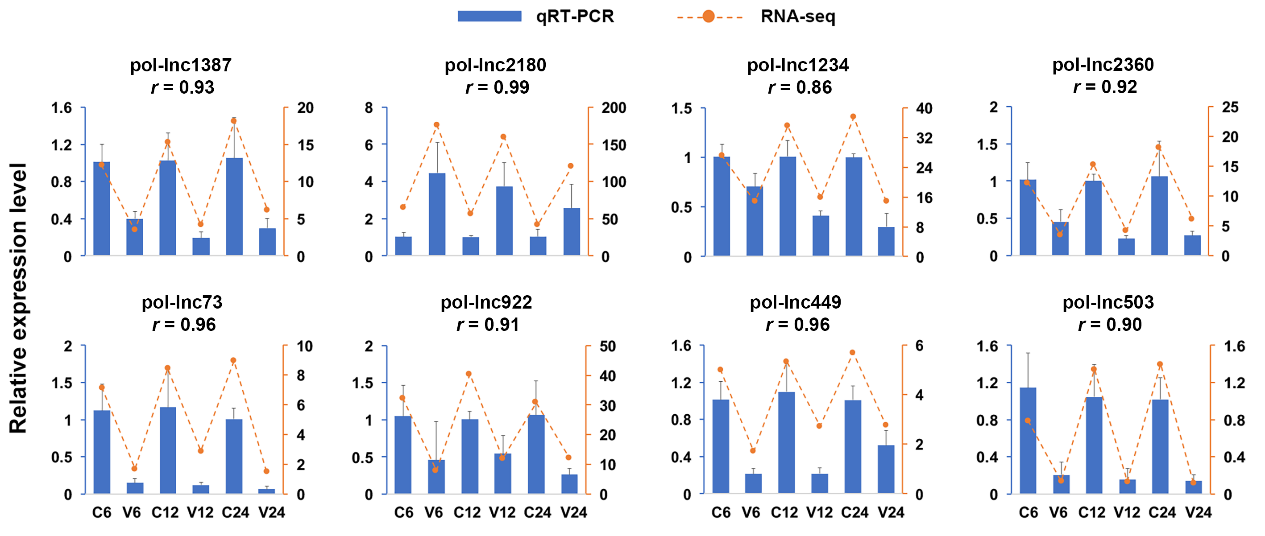

Supplement: Supplementary file 3 — Additional file 3: Figure S1. Validation of DElncRNAs by qRT-PCR. The expression patterns of eight DElncRNAs were tested by qRT-PCR, and the results were compared with that obtained by RNA-sEq. The results are shown as means ± standard deviation (N = 3). Correlations between qRT-PCR and RNA-seq are indicated by correlation coefficient r. [file 12864_2021_7780_MOESM3_ESM.docx]
