# Supplementary material for: Identification and characterization of immune-related lncRNAs and lncRNA-miRNA-mRNA networks of Paralichthys olivaceus involved in Vibrio anguillarum infection
Source: BMC Genomics. 2021 Jun 15;22:447. doi: 10.1186/s12864-021-07780-2 (PMC8204505; doi:10.1186/s12864-021-07780-2)
Supplement: Supplementary file 4 — Additional file 4: Figure S2. Validation of DElncRNA-DETmiR pairs by qRT-PCR. The expression patterns of six pairs of DElncRNA-DETmiR were tested by qRT-PCR. Correlations between DElncRNAs and corresponding DETmiRs are indicated by correlation coefficient r and p values. [file 12864_2021_7780_MOESM4_ESM.docx]

**Fig. S2.** Validation of DElncRNA-DETmiR pairs by qRT-PCR. The expression patterns of six pairs of DElncRNA-DETmiR were tested by qRT-PCR. Correlations between DElncRNAs and corresponding DETmiRs are indicated by correlation coefficient *r* and *p* values.

**
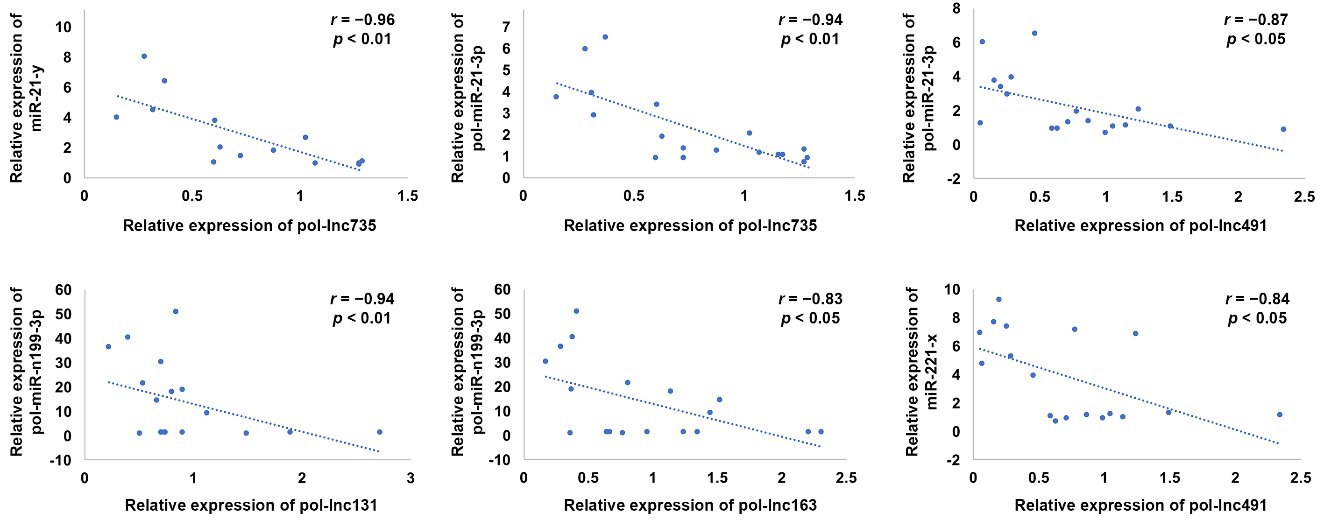
**
