# Supplementary material for: Identification and characterization of immune-related lncRNAs and lncRNA-miRNA-mRNA networks of Paralichthys olivaceus involved in Vibrio anguillarum infection
Source: BMC Genomics. 2021 Jun 15;22:447. doi: 10.1186/s12864-021-07780-2 (PMC8204505; doi:10.1186/s12864-021-07780-2)
Supplement: Supplementary file 5 — Additional file 5: Figure S3. Validation of DETGs by qRT-PCR. The expression patterns of three DETGs involved in the pathway of complement and coagulation cascades were tested by qRT-PCR, and the results were compared with that obtained by RNA-sEq. The results are shown as means ± standard deviation (N = 3). Correlations between qRT-PCR and RNA-seq are indicated by correlation coefficient r. [file 12864_2021_7780_MOESM5_ESM.docx]

**Fig. S3.** Validation of DETGs by qRT-PCR. The expression patterns of three DETGs involved in the pathway of complement and coagulation cascades were tested by qRT-PCR, and the results were compared with that obtained by RNA-seq. The results are shown as means ± standard deviation (N = 3). Correlations between qRT-PCR and RNA-seq are indicated by correlation coefficient *r*.

**
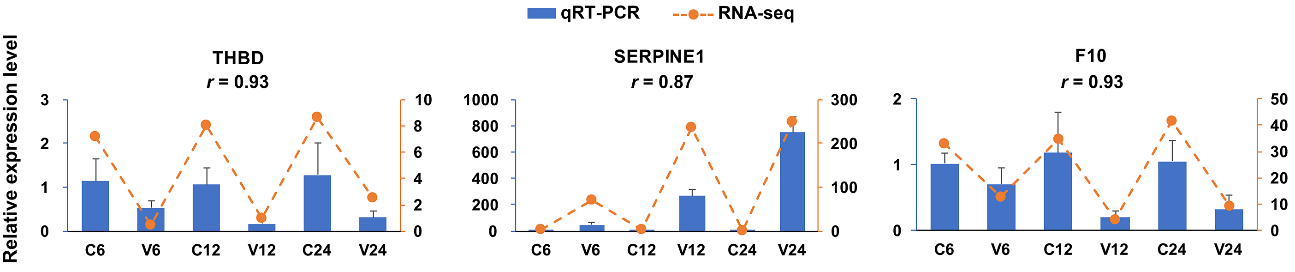
**
